# Optimal adjuvant strategy in intermediate-risk cervical cancer: a systematic review and meta-analysis

**DOI:** 10.1007/s10147-026-03028-9

**Published:** 2026-04-21

**Authors:** Akira Yokoi, Hiroko Machida, Mika Okazawa-Sakai, Koji Nishino, Hanae Sekine, Mizue Itoi, Takumi Mitamura, Fumiaki Isohashi, Keisuke Tsuchida, Haruya Saji, Satoe Fujiwara, Noriko Ii, Miho Watanabe, Tadaaki Nishikawa, Satoshi Nakagawa, Michiko Kodama, Shinya Sato, Kazuhiro Takehara, Tohru Morisada, Shin-ei Noda, Munetaka Takekuma, Hiroaki Kajiyama, Hideki Tokunaga, Tsukasa Baba, Yoichi Kobayashi, Aikou Okamoto

**Affiliations:** 1 Department of Obstetrics and Gynecology, Nagoya University, Aichi, Japan; 2 Department of Obstetrics and Gynecology, Tokai University School of Medicine, Shimokasuya, Isehara, Kanagawa Japan; 3 Department of Gynecologic Oncology, National Hospital Organization Shikoku Cancer Center, Matsuyama, Ehime Japan; 4 Department of Gynecologic Oncology, Niigata Cancer Center Hospital, Niigata, Japan; 5 Department of Obstetrics and Gynecology, Faculty of Medicine, Juntendo, University, Tokyo, Japan; 6 Department of Gynecologic Oncology, Chiba Cancer Center, Chiba, Japan; 7 Department of Obstetrics and Gynecology, Hokkaido University Faculty of Medicine, Hokkaido University, Sapporo, Japan; 8 Department of Radiation Oncology, Nara Medical University, Nara, Japan; 9 Department of Radiation Oncology, Kanagawa Cancer Center, Yokohama, Kanagawa Japan; 10 Department of Gynecology, Kanagawa Cancer Center, Yokohama, Kanagawa Japan; 11 Department of Obstetrics and Gynecology, The University of Osaka Graduate School of Medicine, Osaka, Japan; 12 Department of Radiation Oncology, Ise Red Cross Hospital, Ise-shi, Japan; 13 Department of Diagnostic Radiology and Radiation Oncology, Graduate School of Medicine, Chiba University, Chiba, Japan; 14 Department of Obstetrics and Gynecology, The Jikei University School of Medicine, Tokyo, Japan; 15 Department of Obstetrics and Gynecology, Osaka University, Osaka, Japan; 16 Department of Obstetrics and Gynecology, Faculty of Medicine, Tottori University, Tottori, Japan; 17 Department of Obstetrics and Gynecology, Kyorin University School of Medicine, Tokyo, Japan; 18 Department of Radiation Oncology, International Medical Center, Saitama Medical University, Saitama, Japan; 19 Department of Gynecology, Shizuoka Cancer Center, Shizuoka, Japan; 20 Division of Obstetrics and Gynecology, Faculty of Medicine, Tohoku Medical and Pharmaceutical University, Sendai, Miyagi Japan; 21 Department of Obstetrics and Gynecology, Iwate Medical University, Morioka, Iwate Japan

**Keywords:** Cervical cancer, Systematic review, Intermediate-risk, Survival, Complications

## Abstract

**Objective:**

To evaluate the efficacy and safety of adjuvant treatment strategies following radical hysterectomy for intermediate-risk, early-stage cervical cancer using a reconstructed HR Hazard ratio (HR) meta-analysis.

**Methods:**

A systematic review and meta-analysis was conducted by the Japan Society of Gynecologic Oncology Cervical Cancer Committee. PubMed/MEDLINE, Cochrane, and Ichushi were searched on July 29, 2025, using “cervical cancer,” “intermediate-risk,” and “adjuvant therapy.” Studies comparing adjuvant radiotherapy alone (RT) with no further treatment (NFT), concurrent chemoradiotherapy (CCRT), or systemic chemotherapy (CT) after conventional radical hysterectomy were independently reviewed by two reviewers. Primary outcomes were survival and grade ≥ 3 treatment-related toxicities.

**Results:**

Of 402 screened articles, 24 studies comprising 9278 patients were included: RT (*n* = 4167), NFT (*n* = 2057), CCRT (*n* = 2118), and CT (*n* = 936). The majority of studies enrolled patients with ≥ 2 Sedlis risk factors (median 84.2%, interquartile range 44.7–100%). Compared with NFT, RT significantly improved recurrence-free survival (HR 0.61, *P* < 0.01) but did not confer a significant overall survival benefit (HR 0.77, *P* = 0.09). RT also reduced recurrence in patients with a single risk factor (HR 0.55, *P* < 0.01). RT showed no survival disadvantage compared with CCRT (recurrence-free survival: HR 1.26; overall survival: HR 1.07), and survival outcomes were comparable between RT and CT (recurrence-free survival: HR 0.86; overall survival: HR 1.16) (all *P* > 0.05). Grade ≥ 3 toxicities were significantly lower with RT than with CCRT (odds ratio 0.25; *P* < 0.001).

**Conclusion:**

Adjuvant RT represents an effective and well-tolerated postoperative strategy for intermediate-risk, early-stage cervical cancer. Adjuvant CT may represent a potential alternative option.

**Supplementary Information:**

The online version contains supplementary material available at https://doi.org/10.1007/s10147-026-03028-9.

## Introduction

Cervical cancer remains a major global health burden and is one of the most common malignancies among women worldwide [1]. For patients with early-stage disease, radical hysterectomy with pelvic lymph node assessment remains the cornerstone of primary treatment and yields favorable oncologic outcomes [2] [3]. Nevertheless, disease recurrence occurs in a subset of patients despite optimal surgical management, highlighting the need for appropriate postoperative risk stratification and adjuvant therapy in selected cases [3]. Cisplatin-based concurrent chemoradiotherapy (CCRT) is established as the standard adjuvant treatment for patients with high-risk pathological features following radical surgery, supported by randomized controlled trials demonstrating survival benefits [4].

Postoperative risk stratification in early-stage cervical cancer is primarily based on surgical–pathological factors and classifies patients into high-, intermediate-, and low-risk groups [5]. Intermediate-risk disease is defined by combinations of large tumor size, deep stromal invasion, and lymphovascular space invasion (LVSI). This risk category was initially established in Gynecologic Oncology Group (GOG) studies and subsequently evaluated in the randomized GOG-92 trial, which demonstrated that adjuvant external-beam radiotherapy (RT) significantly reduced recurrence compared with observation in node-negative, parametria-negative stage IB disease with two or more Sedlis criteria [6] [7].

Despite these findings, the optimal postoperative management for intermediate-risk, early-stage cervical cancer remains controversial, and international guidelines provide inconsistent recommendations [5]. Although several systematic reviews and meta-analyses have examined this issue, most have relied on pooled event rates or dichotomous outcomes, limiting assessment of time-dependent oncologic effects [8] [9]. Therefore, this systematic review and meta-analysis incorporated recent trial-era evidence and applied a reconstructed hazard ratio-based approach to evaluate time-to-event outcomes, assessing the efficacy and safety of adjuvant RT alone compared with no further treatment (NFT), systemic chemotherapy (CT), and CCRT following radical hysterectomy.

## Patients and methods

### Literature search and study selection

**Fig. 1:**
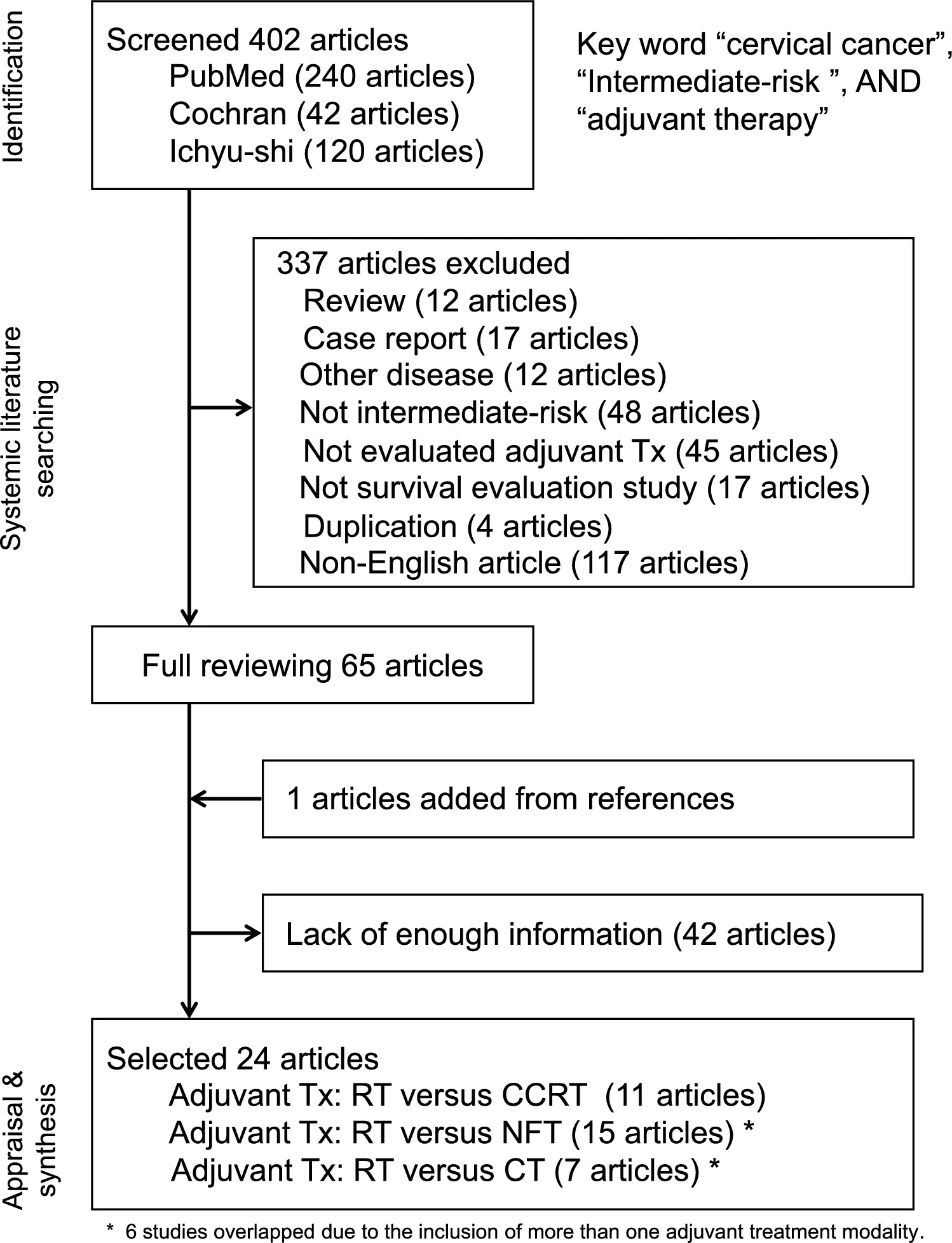
Study schema. *Tx* treatment, *RT*, radiotherapy, *NFT* no further treatment, *CCRT* concurrent chemoradiotherapy, *CT* chemotherapy

The literature search was conducted per the Preferred Reporting Items for Systematic Reviews and Meta-Analyses guidelines [10] using the PubMed/MEDLINE, Cochrane, and Ichushi databases with the keywords “cervical cancer,” “intermediate-risk,” and “adjuvant therapy” on July 29, 2025 (Fig. 1, Supplementary Table S1). Ethical approval was not required for this systematic review and meta-analysis because all data were obtained from previously published peer-reviewed studies. This study was registered in PROSPERO to ensure transparency and methodological rigor (registration number: 1186079).

### Eligibility criteria

Articles based on comparing postoperative adjuvant RT alone with other postoperative adjuvant therapies, including NFT, CCRT, and CT in women with early-stage cervical cancer, were eligible for inclusion. Articles were stratified by postoperative adjuvant treatment strategies for patients with Stage I or II cervical cancer according to the International Federation of Gynecology and Obstetrics staging system. Patients with early-stage cervical cancer underwent radical hysterectomy with or without bilateral salpingo-oophorectomy and nodal evaluation. This study included randomized controlled trials, cohort studies, and case–control series published in the English literature that provided sufficient data on patient demographics, primary treatment, perioperative complications, survival outcomes, and follow-up. Systematic reviews and meta-analyses were excluded from the final analysis and used only for reference screening.

### Data extraction

The references of all the selected articles were reviewed, and articles that met the inclusion criteria were assessed. If multiple publications from the same clinical trial were available, the most recent publication was included. Retrospective interventional or observational studies with substantial cases were selected. Non-case–control trials, single-arm studies in any postoperative adjuvant treatment, systematic reviews, reports on high-risk groups (including nodal metastasis, parametrial involvement, and surgical margin positivity), and basic scientific research were excluded. Additionally, studies involving neoadjuvant CT or RT or with unknown treatment information were excluded.

### Clinical information and definitions

The following variables were extracted from the selected studies: year of publication, study type, country, details of initial treatment (type of radical hysterectomy, adnexal evaluation, or nodal evaluation) and postoperative adjuvant treatments (NFT, RT, CCRT, and CT), details of pathological information (histological subtypes, including squamous cell carcinoma or non-squamous cell carcinoma, LVSI, deep stromal invasion, and tumor size ≥ 4 cm), details of adjuvant treatment-related complications, and recurrence-free and overall survival. Primary treatment was defined as radical hysterectomy with or without bilateral salpingo-oophorectomy and comprehensive nodal evaluation [11]. Adjuvant RT comprised postoperative pelvic external-beam RT. Adjuvant CCRT was defined as RT combined with platinum-based CCRT. Adjuvant CT was administered as a systemic anticytotoxic agent. The proportion of risk factors (≥ 2 factors), including tumor size, LVSI, and deep stromal invasion, meeting the Sedlis criteria [6] was calculated from available data. Recurrence-free survival was defined as the time between the primary treatment and the relapse of cervical cancer. Overall survival indicated the period between disease diagnosis and death from any cause or cancer-specific death. Treatment-related complications were defined as severe adverse events if graded as ≥ 3 according to the National Cancer Institute’s Common Terminology Criteria for Adverse Events [12].

### Statistical analysis

To synthesize survival data, the hazard ratio (HR) and their 95% confidence interval (CI) reported in each study were extracted and pooled after logarithmic transformation. When HRs were not explicitly reported, data were reconstructed from Kaplan–Meier curves using the Tierney method, and a reconstructed HR-based meta-analysis was conducted [13]. Sensitivity analyses were performed to assess the robustness of the pooled estimates by excluding studies in which HRs were reconstructed from Kaplan–Meier curves. If the HR could not be utilized with sufficient power, the number of patients in each treatment arm who experienced an event was compared to estimate the risk ratio (RR) with a 95% CI for dichotomous variables. RR was also used to assess severe adverse events. For continuous variables, the final values and standard deviations were calculated to determine differences in mean values. *P* values < 0.05 were considered statistically significant.

### Data extraction and management

The study protocol was developed in Japanese and conducted as part of a guideline revision project by the Guideline Committee of the Japan Society of Gynecologic Oncology (JSGO). Data were entered into a reference database and extracted independently by three reviewers who were blinded to each other’s assessments (A.Y., H.M., and staff personnel from the Japan Medical Library Association). The quality of the studies was independently assessed by the reviewers (A.Y. and H.M.), and disagreements were resolved through discussion with a third reviewer from the Expert Panel of the JSGO Committee. In cases of missing data or unclear methods, further information was obtained from other published studies on the same trials. For each study, we recorded the detailed methods, study population, sample size, inclusion and exclusion criteria, interventions and comparisons, patient demographics, treatment details, and survival outcomes.

### Assessment of the risk of bias

**Fig. 2:**
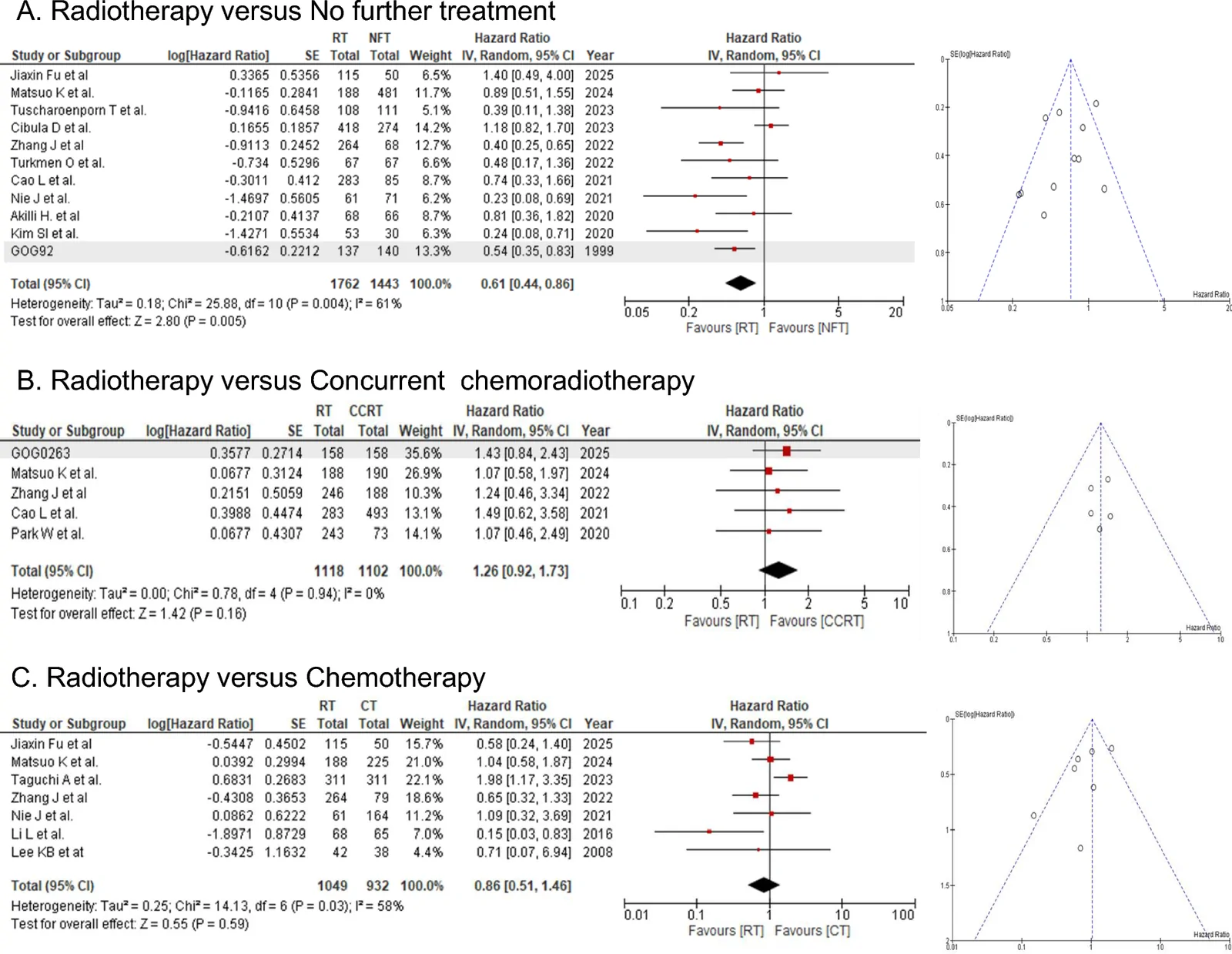
Recurrence-free survival. Forest plot of the meta-analysis and funnel plot for recurrence-free survival based on adjuvant treatment for early-stage cervical cancer

**Fig. 3:**
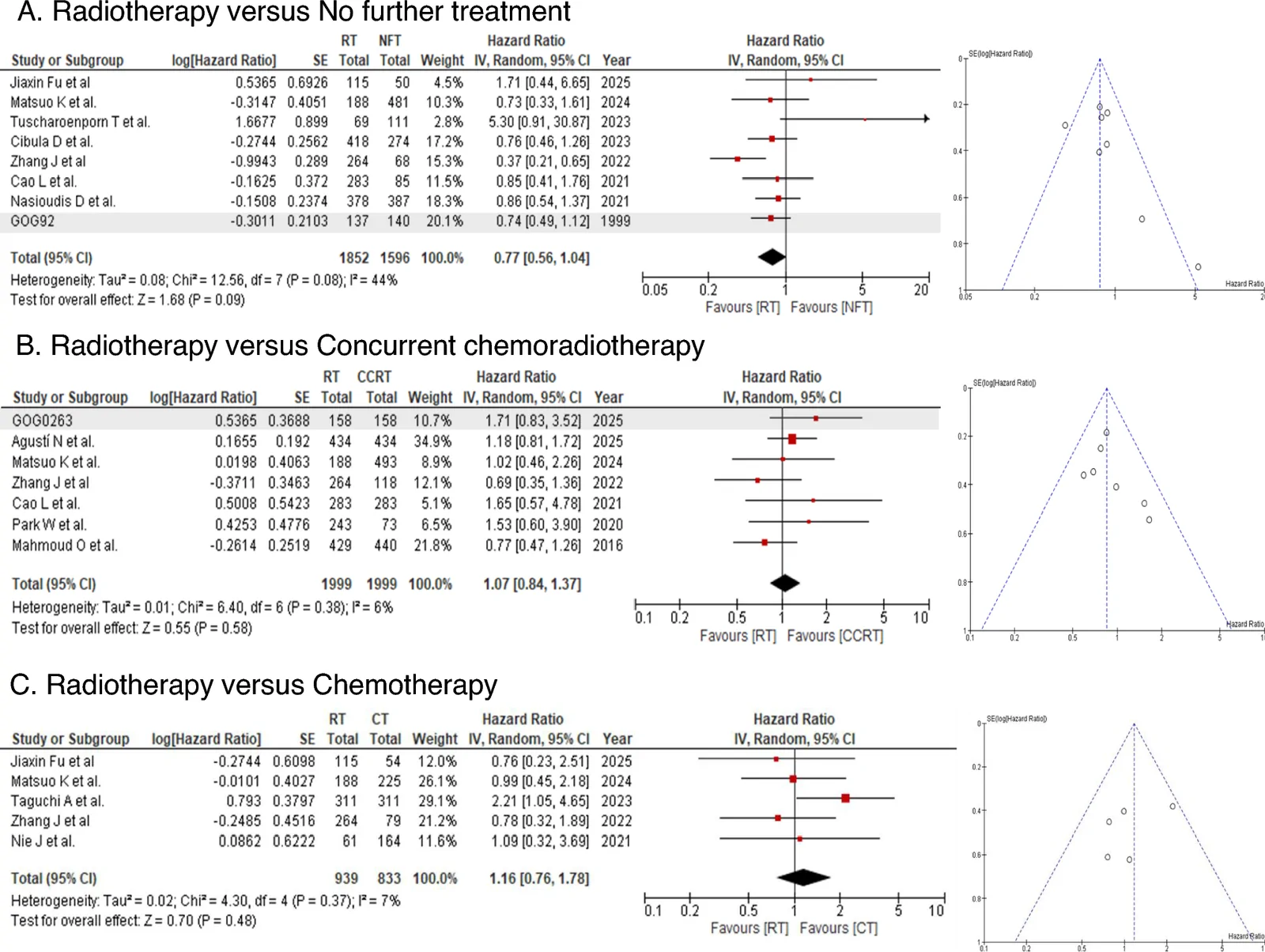
Overall survival. Forest plot of the meta-analysis and funnel plot for overall survival based on adjuvant treatment in early-stage cervical cancer

Using the risk of bias in nonrandomized studies as an intervention tool, the risk of bias was independently assessed by two authors for each study (A.Y. and H.M.). The possible biases included selection, preference, detection, attribution, and reporting (Supplementary Table S2). As blinding the participants or physicians to the assigned treatment was infeasible, blinding (performance and detection biases) was only assessed for outcomes. To investigate publication bias, we performed funnel plot analysis (Figs. 2 and 3).

### Assessment of heterogeneity

HR was used as the measure of association across studies, and Mantel–Haenszel random-effects models were used to calculate summary estimates. Study heterogeneity was assessed through the visual inspection of forest plots and statistical evaluation using Cochran’s *Q* test and the *I*^2^ test. Study data were synthesized to obtain the overall estimates of the treatment effects. Given the clinical heterogeneity among studies, including variations in Sedlis risk factors, surgical radicality, and radiotherapy techniques, a random-effects model was prespecified. The Review Manager software (Version 5.3, Copenhagen: The Nordic Cochrane Centre, The Cochrane Collaboration, 2014) was used. The confidence level in the summary data was examined using the Grading of Recommendations Assessment, Development, and Evaluation for Studies of Interventions and Diagnostic Test Accuracy.

## Results

### Study selection

The literature search initially revealed 402 articles (Fig. 1). Of these, 337 were excluded because they were not case–control studies, did not focus on the target disease or treatment, represented basic research, were duplicates, or were written in languages other than English. After the full-text assessment of the remaining 65 articles, one additional article was identified through reference screening. Twenty-four studies met the eligibility criteria [6] 7, [14–36], comprising 2 and 22 prospective and retrospective studies, respectively. The data included outcomes for RT (*n* = 4167), NFT (*n* = 2057), CCRT (*n* = 2118), and CT (*n* = 932) in 24, 15, 11, and 7 studies, respectively, among patients with intermediate-risk early-stage cervical cancer.

### Risk of bias of included studies

Except for two prospective randomized controlled trials (RCTs), the included studies exhibited a moderate-to-serious risk of bias (Supplementary Table 1). Publication bias was evaluated using funnel plots (Figs. 2 and 3). As expected for cohort studies, several articles [14–16] did not provide sufficient information regarding the assessment of confounding factors or baseline differences between the compared groups.

### Study characteristics

The characteristics of the included studies are summarized in Table 1. Most of the studies were published in 2010 or later (84.0%) and were Asian reports (68.0%). A total of 9274 patients were analyzed, including 4167 (44.9%) who received RT, 2057 (22.2%) who did not receive adjuvant therapy, 2118 (22.8%) who had CCRT, and 932 (10.0%) who underwent CT. Across all studies, the proportion of patients with ≥ 2 intermediate-risk factors had a median of 84.2% (interquartile range [IQR], 43.7–100). The prevalence of individual risk factors had a median of 28.6% (IQR, 20.3–32.0) for non-squamous cell carcinoma histology, 59.1% (43.6–71.5) for LVSI, 78.3% (61.3–83.0) for deep stromal invasion, and 38.0% (22.9–58.0) for tumor size ≥ 4 cm. The proportions of patients with ≥ 2 risk factors were similar among treatment comparisons: 76.0% (IQR, 43.5–100) for RT vs. NFT, 100% (57.2–100) for RT vs. CCRT, and 44.3% (0.0–92.3) for RT vs. CT (*P* = 0.21). In addition, the follow-up duration was comparable between RT and the other treatment groups, including NFT, CCRT, and CT (median 56.4 vs. 50.9 months, *P* = 0.78).

**Table 1:** Characteristics of the studies

Author, year	Study type	Country	Study population									
			No.RT	No.Other Tx	TotalNo	Risk factor ≥ 2*	Non-SCC	LVSI Present	Deep SI	Size ≥ 4 cm	Follow-up (*m*) RT Other Tx	
Radiotherapy vs no further treatment				NFT								
GOG92. 1999/2006 42, 43	RCT	USA	137	140	277	100%**	21.3%	70.4%	75.8%	26.7%	60.0	60.0
Jiaxin F et al. 2025 44	Retro	China	115	50	219	0%	100%	5.5%	80.8%	13.7%	50.0	55.0
Matsuo K et al. 2024 [34]	Retro	Japan	188	481	1084	92.3%	39.4%	73.8%	60.0%	81.0%	67.2	67.2
Tuscharoenporn T et al. 2023[33]	Retro	Thailand	108	111	219	100%**	28.3%	90.9%	84.0%	na	76.1	95.4
Cibula D et al. 2023[2]	Retro	International	418	274	692	76.0%^+^	29.1%	76.0%	na	42.6%	56.4	51.6
Turkmen O et al. 2022[30]	Retro	Turkey	67	67	183	60.1%	23%	57.9%	82.0%	33.9%	48.0	48.0
Zhang J et al. 2022[29]	Retro	China	264	68	976	23.6%	15.7%	9.2%	37.3%	34.4%	49.0	48.0
Nasioudis D et al. 2021[27]	Retro	USA	378	387	765	100%**	31.5%	65.6%	na	50.2%	45.0	44.8
Cao L et al. 2021[26]	Retro	China	283	85	861	100%**	0%	46.5%	48.7%	75.2%	64.0	62.0
Nie J et al. 2021[28]	Retro	China	61	71	571	44.3%	15.1%	52.4%	75.7%	22.9%	62.0	62.0
Kim SI et al. 2020[25]	Retro	Korea	53	30	83	100%**	16.8%	60.2%	78.3%	71.1%	40.4	40.4
Akilli H et al. 2020[24]	Retro	Turkey	68	66	134	100%**	17.9%	73.9%	86.3%	33.6	54.0	51.0
Nakamura K et al. 2016[21]	Retro	Japan	19	46	75	33.3%	32.0%	42.7%	61.3%	0%	90.6	78.7
Ryu SY et al. 2011[18]	Retro	Korea	49	34	172	40.7%	24.9%	11.4%	80.0%	na	44.6	44.6
Ayhan A et al. 2004[15]	Retro	Turkey	69	147	216	52.3%	na	71.8%	73.1%	5.1%	31.2	31.2
Radiotherapy vs concurrent chemoradiotherapy				CCRT								
GOG0263. 2025 45	RCT	Korea	158	158	316	100%	16.3%	72.7%	81.6%	na	76.5	76.5
Agustí N et al. 2025[35]	Retro	USA	434	434	868	100%**	40.0%	69.1%	na	57.1%	64.2	66.0
Matsuo K et al. 2024[34]	Retro	Japan	188	190	1084	92.3%	39.4%	73.8%	60.0%	81.0%	67.2	67.2
Zhang J et al. 2022[29]	Retro	China	264	118	529	43.5%	28.8%	17.0%	68.8%	63.5%	49.0	49.0
Cao L et al. 2021[26]	Retro	China	283	493	861	100%**	0%	46.5%	48.7%	75.2%	64.0	62.0
Park W et al. 2020[23]	Retro	Korea	243	73	316	70.9%	26.6%	46.2%	88.0%	50.9%	70.0	70.0
Mahmoud O et al. 2016[22]	Retro	USA	429	440	869	100%**	37.0%	32.0%	na	58.0%	48.0	48.0
Nakamura K et al. 2016[21]	Retro	Japan	10	14	75	33.3%	32.0%	42.7%	61.3%	0%	90.6	98.0
Song S et al. 2012[19]	Retro	Korea	56	54	110	100%	21.8%	63.6%	93.6%	na	75.6	48.0
Ryu SY et al. 2011[18]	Retro	Korea	49	89	172	40.7%	24.9%	11.4%	80.0%	na	44.6	44.6
Kim K et al. 2009[25]	Retro	Korea	24	55	79	na	29.1%	49.4%	91.1%	38.0%	54.0	48.0
Radiotherapy vs chemotherapy				CT								
Jiaxin F et al. 2025	Retro	China	115	50	219	0%	100%	5.5%	80.8%	13.7%	50.0	55.0
Matsuo K et al. 2024[34]	Retro	Japan	188	225	1084	92.3%	39.4%	73.8%	60.0%	81.0%	67.2	67.2
Taguchi A et al. 2023[31]	Retro	Japan	311	311	960	62%	32.0%	62.9%	57.6%	25.0%	63.0	63.0
Zhang J et al. 2022[29]	Retro	China	264	79	976	23.6%	15.7%	9.2%	37.3%	34.4%	49.0	49.0
Nie J et al. 2021[28]	Retro	China	61	164	571	44.3%	15.1%	52.4%	75.7%	22.9%	62.0	62.0
Li L et al. 2016[20]	Retro	China	68	65	133	0%	8.3%	na	24.8%	na	36.4	30.8
Lee KB et al. 2008[17]	Retro	Korea	42	38	80	100%	20.0%	48.8%	78.8%	na	56.4	40.7

^*^Risk factors were defined by the Sedlis criteria; non-SCC was reported but not counted

^**^The number of risk factors was not specified, though definitions followed the Sedlis criteria

*No* number, *RT* radiotherapy, *Tx* treatment, *NFT* no further treatment, *CCRT*, concurrent chemoradiotherapy, *CT* chemotherapy, *SCC* Squamous cell carcinoma, *LVSI* lymph vascular invasion, *SI* stromal invasion, *na* not applicable, (*m*) month, *Retro* retrospective study, *RCT* randomized controlled trial

### Synthesis of results

#### Survival, recurrence, and toxicity outcomes: RT versus NFT

Our study conducted a meta-analysis of the data from one RCT and 14 retrospective studies between the RT and NFT groups (*n* = 4334) (Table 1). Adjuvant RT was associated with a significantly improved recurrence-free survival compared with that under NFT (HR 0.61, 95% CI 0.44–0.86, *P* = 0.005, Fig. 2). Although the overall survival also tended to favor the RT group, the difference was not statistically significant (HR 0.77, 95% CI 0.56–1.04; *P* = 0.09, Fig. 3). Furthermore, among patients with a single risk factor, adjuvant RT significantly reduced recurrence compared with that under NFT (recurrence-free survival: HR 0.55, 95% CI 0.31–0.97, *P* = 0.04, Supplementary Fig. S1). Sensitivity analyses yielded similar results (recurrence-free survival: HR 0.59, 95% CI 0.44–0.79; overall survival: HR 0.76, 95% CI 0.52–1.11). Recurrence patterns were evaluated based on reported event counts, and RRs were calculated because time-to-event data, including HRs or Kaplan–Meier estimates, were not available (Supplementary Fig. S2). Local recurrence was less frequent in the RT group than in the NFT group (8.3% vs. 12.2%; RR 0.75, 95% CI 0.56–0.99; *P* = 0.04), whereas distant recurrence rates were comparable between the groups (6.3% vs. 4.8%; RR 1.04, 95% CI 0.47–2.30, *P* = 0.93). Sever adverse events associated with adjuvant treatment were reported in five studies (Supplementary Fig. S3). The incidence of severe adverse events was significantly higher in the RT group than in the NFT group (6.5% vs. 1.9%; RR 5.17, 95% CI 2.70–9.90, *P* < 0.001). The most common severe adverse events were genitourinary (6.3%), gastrointestinal (3.2%), and hematologic (1.6%) toxicities.

#### Survival, recurrence, and toxicity outcomes: RT versus CCRT

For the RT and CCRT groups (*n* = 4256), our study conducted a meta-analysis of the data from one RCT and 10 retrospective studies (Table 1). Recurrence-free survival showed no significant differences between the RT and CCRT groups (HR 1.26, 95% CI 0.92–1.73, *P* = 0.16, Fig. 2). In addition, overall survival did not differ significantly between the groups (HR 1.07, 95% CI 0.84–1.37, *P* = 0.58, Fig. 3). Recurrence patterns were evaluated based on reported events in five studies (Supplementary Fig. S2). Local recurrence rates were comparable between the RT and CCRT groups (5.4% vs. 2.7%; RR 2.13, 95% CI 0.89–5.07, *P* = 0.09). In contrast, distant recurrence tended to occur more frequently in the RT group than in the CCRT group (9.5% vs. 6.2%; RR 1.48, 95% CI 1.04–2.10, *P* = 0.03). Severe adverse events related to adjuvant treatment were reported in five studies (Supplementary Fig. S3). The incidence of severe adverse events was significantly lower in the RT group than in the CCRT group (11.1% vs. 38.7%; RR 0.28, 95% CI 0.17–0.44, *P* < 0.001). The most common severe adverse events were hematologic toxicities (RT vs. CCRT: 6.0% vs. 33.6%), followed by gastrointestinal (3.9% vs. 7.4%) and genitourinary (2.0% vs. 3.0%) toxicities.

#### Survival, recurrence, and toxicity outcomes: RT versus CT

Moreover, our study compared the outcomes between RT and CT groups (*n* = 1981). The meta-analysis of data from seven retrospective studies is shown in Table 1. Recurrence-free survival exhibited no significant differences between the RT and CT groups (HR 0.86, 95% CI 0.51–1.46, *P* = 0.59, Fig. 2). In addition, overall survival showed no significant differences between the two groups (HR 1.16, 95% CI 0.76–1.78, *P* = 0.48, Fig. 3). Recurrence patterns were evaluated based on the reported events in only two studies (Supplementary Fig. S2). Local and distant recurrence rates were comparable between the RT and CT groups (Local: 0.9% vs. 3.8%; RR 0.49, 95% CI 0.02–13.13, *P* = 0.67; Distant: 2.7% vs. 3.8%; RR 0.69, 95% CI 0.16–2.98, *P* = 0.62). Only one study reported events related to adjuvant treatment. The most common severe adverse event was hematological toxicity in the CT group (RT vs. CT: 27.9% vs. 53.8%), whereas gastrointestinal (4.4% vs. 0%) and genitourinary (5.9% vs. 0%) toxicities were more frequent in the RT group.

## Discussion

### Summary of main results

This systematic review and meta-analysis evaluated adjuvant treatment strategies for patients with intermediate-risk, early-stage cervical cancer following radical hysterectomy and pelvic lymphadenectomy. The results indicate that postoperative RT significantly reduces recurrence compared with NFT, whereas CCRT does not provide additional benefit over RT alone. Notably, RT was associated with reduced recurrence even among patients with a single intermediate-risk factor, underscoring its role as a fundamental adjuvant modality in this setting. Systemic CT demonstrated recurrence outcomes comparable to RT, suggesting a potential alternative approach in selected patients. However, the lack of a clear overall survival benefit across treatment strategies highlights the importance of balancing oncologic efficacy against treatment-related morbidity when selecting adjuvant therapy.

### Results in the context of published literature

A major challenge highlighted in our review is the inconsistent definition of the intermediate-risk category across geographical regions and guideline frameworks [37]. Although the Sedlis criteria remain acceptable [6], several contemporary models incorporate different thresholds for tumor size, various grading systems for LVSI, and different methodologies in defining deep stromal invasion. These discrepancies hinder direct comparisons across studies and may contribute to conflicting results regarding the effectiveness of treatment intensification. Therefore, the standardization of definitions, ideally supported by prospective validation, is crucial to optimizing the generalizability of future evidence.

Another key finding was that intermediate-risk features did not confer uniform biological or prognostic significance [37]. Factors such as extensive LVSI and deeply invasive adenocarcinoma were associated with a markedly higher risk of recurrence than tumor size alone [5, 37], indicating substantial heterogeneity within the intermediate-risk category. This suggests that the current classification may be overly broad and that refined risk assessment incorporating quantitative deep stromal invasion and structured LVSI scoring may improve patient selection for systemic therapy [5]. Furthermore, given that histopathological subtype influences postoperative treatment responsiveness [38, 39], histology-based subgroup analyses should be incorporated into future studies to facilitate more tailored therapeutic strategies.

The evolving landscape of radiation delivery complicates the interpretation of historical data, as many landmark trials predated the widespread use of intensity-modulated radiotherapy (IMRT), which reduces toxicity without compromising local control [38]. Accordingly, the efficacy of adjuvant radiotherapy should be interpreted in the context of modern radiotherapy practice. Notably, the NRG Oncology/GOG-263 trial conducted in the IMRT era did not demonstrate a survival benefit from the addition of cisplatin-based chemotherapy, suggesting that the incremental value of postoperative CCRT may be limited, particularly in intermediate-risk populations.

Systemic treatment options for cervical cancer are rapidly evolving [40]. Particularly, immune checkpoint inhibitors have transformed the management of recurrent and metastatic disease, and ongoing trials are exploring their use in earlier treatment settings [40]. In the present study, adjuvant CT for early-stage, intermediate-risk cervical cancer resulted in survival outcomes comparable to postoperative RT, suggesting its feasibility as a postoperative option. This finding may be especially relevant in Japan, where a higher prevalence of underweight young women may increase susceptibility to radiation-related toxicity [17] [41]. The ongoing RCT (JGOG1082) is evaluating adjuvant CT as an alternative to RT in high-risk early-stage cervical cancer, with potential implications for intermediate-risk disease.

### Strengths and weaknesses

The primary strength of this study lies in its consideration of an HR-based meta-analysis rather than a simple pooled analysis of survival data derived from event counts and total sample sizes, as previously reported. To synthesize survival data, the HRs and their 95% CIs reported in each study were extracted and pooled after logarithmic transformation. This approach enabled us to apply an appropriate strategy to synthesize time-to-event outcomes.

However, several limitations should be acknowledged. First, although this meta-analysis included 24 studies, only two were randomized controlled trials, which may limit methodological rigor and introduce potential bias (Supplemental Table S1). The predominance of retrospective and nonrandomized studies increases the risk of selection bias, confounding, and unmeasured differences in baseline characteristics, which may have influenced treatment selection and survival outcomes. In addition, variability in study design, patient selection, and outcome definitions may have introduced both clinical and methodological heterogeneity, potentially affecting the magnitude and direction of the pooled estimates. Although statistical methods, including the use of a random-effects model, were applied to account for between-study variability, residual bias cannot be excluded, and the findings should therefore be interpreted with caution. Furthermore, although the Sedlis criteria were widely applied, the specific risk factors varied across studies. Detailed information on histological subtypes, surgical procedures, radiotherapy techniques (including intensity-modulated radiotherapy [IMRT]), and chemotherapy regimens was often insufficiently reported, precluding stratified analyses.

## Conclusions

This systematic review and meta-analysis indicates that adjuvant RT is preferable to NFT for patients with intermediate-risk, early-stage cervical cancer. CCRT did not demonstrate clear superiority over RT alone, while systemic CT may represent a potential alternative, although its role remains uncertain. These findings support postoperative RT as the current cornerstone of adjuvant management, and routine de-escalation to observation alone should be avoided in the absence of clearly favorable biological features.

## Supplementary Information

Below is the link to the electronic supplementary material.

Supplementary file1 (DOCX 303 KB)

## Data Availability

In accordance with the journal’s guidelines, we will provide our data for independent analysis by a selected team from the Editorial Team for the purposes of additional data analysis or for the reproducibility of this study in other centers if such is requested.

## Declarations

### Clinical trail registry

Registration number: 1186079 registration date: 7 November 2025.
