# Supplementary material for: Optimal adjuvant strategy in intermediate-risk cervical cancer: a systematic review and meta-analysis
Source: Int J Clin Oncol. 2026 Apr 21;31(6):1084–95. doi: 10.1007/s10147-026-03028-9 (PMC13201331; doi:10.1007/s10147-026-03028-9)
Supplement: Supplementary file 1 — Supplementary file1 (DOCX 303 KB) [file 10147_2026_3028_MOESM1_ESM.docx]

Supplemental Figure 1. Recurrence-free survival


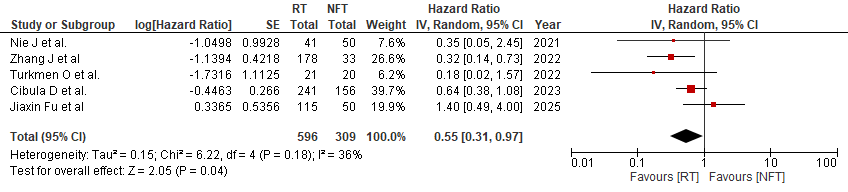
A. Radiotherapy versus No further treatment in single risk factor

Supplementary Figure S2. Recurrence site

A. Radiotherapy versus No further treatment in local recurrence


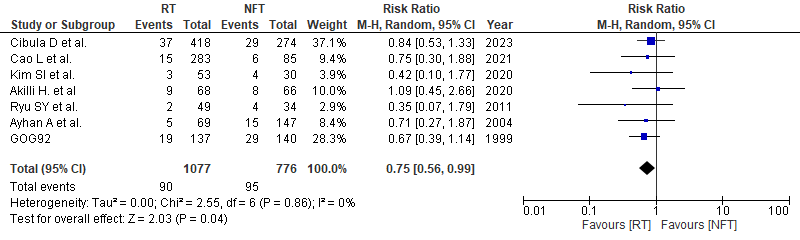

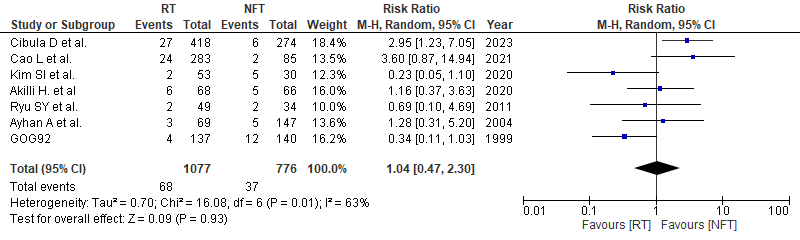


B. Radiotherapy versus No further treatment in distant recurrence


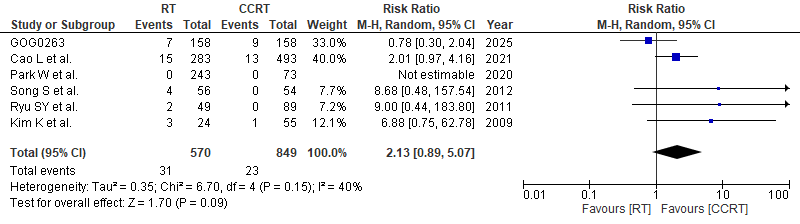


C. Radiotherapy vs Concurrent chemoradiotherapy in local recurrence


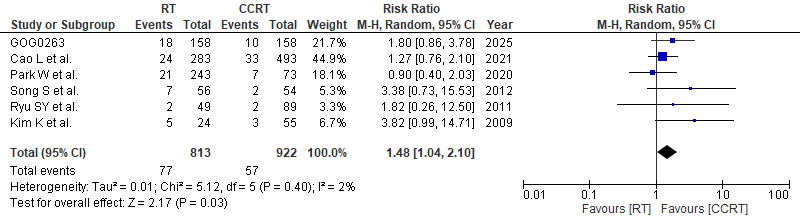


D. Radiotherapy vs Concurrent chemoradiotherapy in distant recurrence


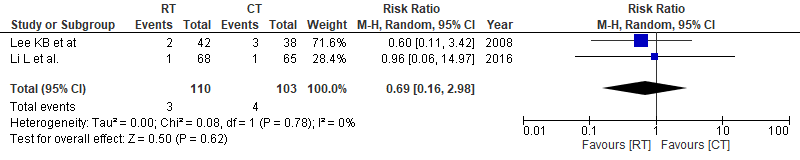

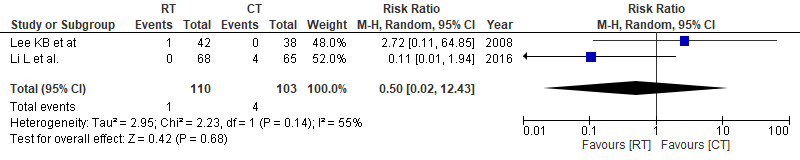


E. Radiotherapy vs Chemotherapy in local recurrence

F. Radiotherapy vs Chemotherapy in distant recurrence


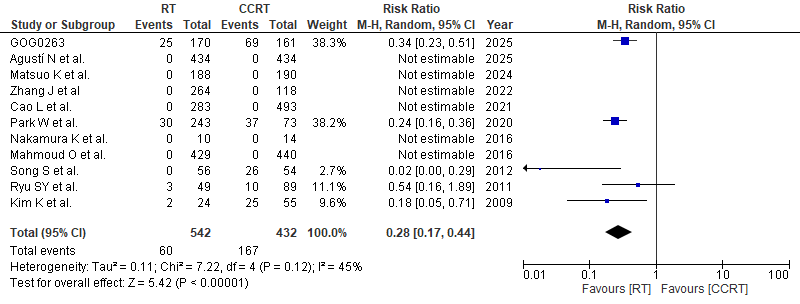


Supplementary Figure S3. Sever adverse events

C. Radiotherapy versus Chemotherapy

Insufficient data were obtained.

B. Radiotherapy versus Concurrent chemoradiotherapy

A. Radiotherapy versus No further treatment


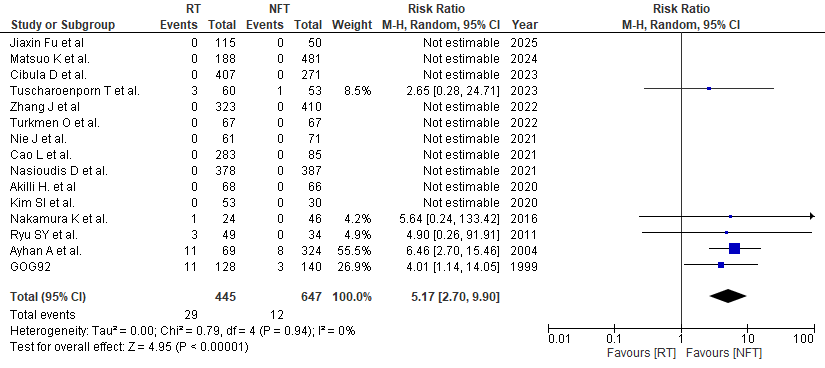


Supplemental Table S1 The detailed search strategy for each database

PubMed　 Date of search：2025/7/29（Thu）

|  | |  |
| --- | --- | --- |
| No. | Search query | Number of articles |
| #01 | "Uterine Cervical Neoplasms/therapy"[Mesh] | 41,239 |
| #02 | "Chemotherapy, Adjuvant"[Mesh] OR "Radiotherapy, Adjuvant"[Mesh] OR "Chemoradiotherapy, Adjuvant"[Mesh] | 68,717 |
| #03 | "Neoplasm Recurrence, Local"[Mesh] OR "Risk"[Mesh] | 1,624,338 |
| #04 | "Tumor Burden"[Mesh] OR "Neoplasm Invasiveness"[Mesh] OR "Lymphatic Metastasis"[Mesh] OR "intermediate risk"[TIAB:~3] | 231,949 |
| #05 | #1 AND #2 AND #3 AND #4 | 194 |
| #06 | ("cervical cancer"[TI] OR "cervix cancer"[TI:~4] OR "cervical neoplasm*"[TI] OR "cervical carcinoma"[TI]) AND (hysterectomy[TIAB] OR surg*[TIAB]) AND risk[TIAB] AND (intermediate[TIAB] OR "tumor size"[TIAB] OR "lymphovascular space invasion"[TIAB] OR LVSI[TIAB] OR "lymph-vascular invasion"[TIAB] OR "deep stromal invasion"[TIAB:~4]) AND ("adjuvant therapy"[TIAB:~4] OR "adjuvant treatment"[TIAB:~4] OR "postoperative irradiation"[TIAB:~4]) | 158 |
| #07 | #5 OR #6 | 316 |
| #08 | #7 AND ("Meta-Analysis"[PT] OR "Meta-Analysis as Topic"[Mesh] OR "meta-analysis"[TIAB]) | 13 |
| #09 | #7 AND ("Cochrane Database Syst Rev"[TA] OR "Systematic Review"[PT] OR "Systematic Reviews as Topic"[Mesh] OR "systematic review"[TIAB]) | 10 |
| #10 | #7 AND ("Practice Guideline"[PT] OR "Practice Guidelines as Topic"[Mesh] OR "Consensus"[Mesh] OR "Consensus Development Conferences as Topic"[Mesh] OR "Consensus Development Conference"[PT] OR guideline*[TI] OR consensus[TI]) | 2 |
| #11 | #8 OR #9 OR #10 | 17 |
| #12 | #7 AND ("Randomized Controlled Trial"[PT] OR "Randomized Controlled Trials as Topic"[Mesh] OR (random*[TIAB] NOT medline[SB])) | 23 |
| #13 | #7 AND ("Clinical Trial"[PT] OR "Clinical Trials as Topic"[Mesh] OR "Observational Study"[PT] OR "Observational Studies as Topic"[Mesh] OR ((clinical trial*[TIAB] OR case control*[TIAB] OR case comparison*[TIAB]) NOT medline[SB])) | 38 |
| #14 | (#12 OR #13) NOT #11 | 37 |
| #15 | #7 AND ("Epidemiologic Methods"[Mesh] OR "Comparative Study"[PT] OR "Multicenter Study"[PT] OR "Validation Study"[PT] OR "Evaluation Study"[PT] OR (("survival analysis"[TIAB] OR cohort*[TIAB] OR comparative stud*[TIAB] OR follow-up stud*[TIAB] OR prospective stud*[TIAB] OR retrospective study*[TIAB]) NOT medline[SB])) | 285 |
| #16 | #15 NOT (#11 OR #14) | 240 |

Cochrane database　Date of search：2025/7/29（Thu）

| No. | Search query | Number of articles |
| --- | --- | --- |
| #01 | "cervical cancer":ti OR "cervix cancer":ti OR "cervical neoplasm":ti OR "cervical carcinoma":ti | 3,504 |
| #02 | hysterectomy:ti,ab,kw OR surg*:ti,ab,kw | 363,708 |
| #03 | risk:ti,ab,kw AND (intermediate:ti,ab,kw OR "tumor size":ti,ab,kw OR "lymphovascular space invasion":ti,ab,kw OR LVSI:ti,ab,kw OR "lymph-vascular invasion":ti,ab,kw OR "deep stromal invasion":ti,ab,kw) | 10,117 |
| #04 | (adjuvant:ti,ab,kw OR postoperative:ti,ab,kw) AND (therapy:ti,ab,kw OR chemotherapy:ti,ab,kw OR radiotherapy:ti,ab,kw OR irradiation:ti,ab,kw) | 91,759 |
| #05 | #1 AND #2 AND #3 AND #4 | 43 |
| #06 | #5 CDSR | 1 |
| #07 | #5 CCRCT | 42 |

Ichushi　Date of search：2025/7/29（Thu）

| No. | Search query | Number of articles |
| --- | --- | --- |
| #01 | 子宮頸部腫瘍/TH | 36,001 |
| #02 | アジュバント化学療法/TH or アジュバント放射線療法/TH or アジュバント放射線化学療法/TH | 47,391 |
| #03 | 腫瘍再発/TH or リスク/TH | 310,394 |
| #04 | 腫瘍量/TH or 腫瘍侵入性/TH or リンパ行性転移/TH | 127,388 |
| #05 | #1 and #2 and #3 and #4 | 52 |
| #06 | (子宮頸部腫瘍/TI or 子宮頸癌/TI or 子宮頚部がん/TI or 子宮頚部ガン/TI or 子宮頸がん/TI or 子宮頸ガン/TI or 子宮頸部がん/TI or 子宮頸部ガン/TI or 子宮頸部癌/TI or "cervical cancer"/TI or "cervix cancer"/TI or "cervical neoplasm"/TI or "cervical carcinoma"/TI) and (術後/TA or 補助/TA or アジュバント/TA or postoperative/TA or adjuvant/TA) and (治療/TA or 療法/TA or 照射/TA or therapy/TA or treatment/TA or chemotherapy/TA or radiotherapy/TA or irradiation/TA) and (腫瘍量/TA or 中リスク/TA or 中間リスク/TA or 腫瘍サイズ/TA or 腫瘍体積/TA or 腫瘍容積/TA or 腫瘍容量/TA or 浸潤/TA or 深達度/TA or 侵襲性/TA or 侵入性/TA or "lymphovascular space invasion"/TA or "deep stromal invasion"/TA or intermediate/TA) | 215 |
| #07 | #5 or #6 | 259 |
| #08 | #7 and (メタアナリシス/TH or システマティックレビュー/TH or 診療ガイドライン/TH) | 2 |
| #09 | #7 and (RD=メタアナリシス,診療ガイドライン) | 1 |
| #10 | #7 and (メタアナリシス/TA or システマティックレビュー/TA or 診療ガイドライン/TA) | 0 |
| #11 | #8 or #9 or #10 | 2 |
| #12 | #7 and (介入研究/TH or 疫学研究特性/TH or 疫学的研究デザイン/TH) | 61 |
| #13 | #7 and (RD=ランダム化比較試験,準ランダム化比較試験,比較研究) | 51 |
| #14 | #7 and (介入研究/TA or 臨床試験/TA or ランダム化/TA or 無作為化/TA or 非劣性試験/TA or 同等性試験/TA or ランダム割付け/TA or 疫学研究/TA or 疫学的研究/TA or 観察研究/TA or 縦断研究/TA or 後向き研究/TA or 症例対照研究/TA or 前向き研究/TA or コホート研究/TA or 追跡研究/TA or 断面研究/TA or 介入研究/TA or 実現可能性研究/TA or 双生児研究/TA or 多施設共同研究/TA or パイロットプロジェクト/TA or 標本調査/TA or 臨床試験/TA or 第I相試験/TA or 第II相試験/TA or 第III相試験/TA or 第IV相試験/TA or 第1相試験/TA or 第2相試験/TA or 第3相試験/TA or 第4相試験/TA or 第一相試験/TA or 第二相試験/TA or 第三相試験/TA or 第四相試験/TA or クロスオーバー研究/TA) | 15 |
| #15 | (#12 or #13 or #14) not #11 | 71 |
| #16 | #7 and (PT=原著論文,総説 CK=ヒト) | 182 |
| #17 | #16 not (#11 or #15) | 120 |

Supplemental Table 2. Risk of bias evaluation

|  | | Selection bias | | Preference bias | Detection bias | Attribution bias | Reporting bias | | The overall risk of bias | |
| --- | --- | --- | --- | --- | --- | --- | --- | --- | --- | --- |
| Author, year | Study type | Confounding | Selection bias | Classification of intervention | Deviations from intended interventions | Missing data | Measurement of outcomes | Selection of reported results | |  |
| Observation versus Radiotherapy | | |  |  |  |  |  |  | |  |
| GOG-92. 1999/2006 | Prospective | Low | Low | Low | Low–Moderate | Low | Low | Low | | Low |
| Jiaxin Fu et al. 2025 | Retrospective | Moderate | Serious | Moderate | Moderate | Serious | Serious | Serious | | Moderate |
| Matsuo K, et al. 2024 | Retrospective | Moderate | Moderate | Moderate | Moderate | Moderate | Moderate | Moderate | | Moderate |
| Tuscharoenporn T et al. 2023 | Retrospective | Serious | Serious | Moderate | Moderate | Serious | Serious | Serious | | Serious |
| Cibula D et al. 2023 | Retrospective | Moderate | Moderate | Moderate | Moderate | Moderate | Moderate | Moderate | | Moderate |
| Turkmen O et al. 2022 | Retrospective | Serious | Serious | Moderate | Serious | Serious | Moderate | Moderate | | Serious |
| Zhang J et al. 2022 | Retrospective | Serious | Serious | Moderate | Serious | Serious | Moderate | Moderate | | Moderate |
| Nasioudis D et al. 2021 | Retrospective | Serious | Moderate | Moderate | Low–Moderate | Moderate | Moderate | Moderate | | Moderate |
| Cao L et al. 2021 | Retrospective | Serious | Serious | Moderate | Moderate | Serious | Moderate | Moderate | | Moderate |
| Nie J et al. 2021 | Retrospective | Serious | Serious | Serious | Moderate | Serious | Serious | Serious | | Serious |
| Kim SI et al. 2020 | Retrospective | Serious | Serious | Moderate | Moderate | Serious | Moderate | Moderate | | Serious |
| Akilli H et al. 2020 | Retrospective | Serious | Serious | Moderate | Moderate | Serious | Moderate | Moderate | | Serious |
| Nakamura K et al. 2016 | Retrospective | Serious | Serious | Serious | Moderate | Serious | Serious | Serious | | Serious |
| Ryu SY et al. 2011 | Retrospective | Serious | Serious | Moderate | Serious | Serious | Moderate | Moderate | | Serious |
| Ayhan A et al. 2004 | Retrospective | Serious | Serious | Moderate | Moderate | Serious | Moderate | Moderate | | Serious |
| Radiotherapy versus Concurrent Chemoradiotherapy | | |  |  |  |  |  |  | |  |
| GOG-0263. 2025 | Prospective | Low | Moderate | Low | Moderate | Low | Low | Low | | Low |
| Agustí N et al. 2025 | Retrospective | Moderate | Moderate | Moderate | Moderate | Moderate | Moderate | Moderate | | Moderate |
| Matsuo K, et al. 2024 | Retrospective | Moderate | Moderate | Moderate | Moderate | Moderate | Moderate | Moderate | | Moderate |
| Zhang J et al. 2022 | Retrospective | Serious | Serious | Moderate | Serious | Serious | Moderate | Moderate | | Moderate |
| Cao L et al. 2021 | Retrospective | Serious | Serious | Serious | Moderate | Serious | Moderate | Moderate | | Moderate |
| Park W et al. 2020 | Retrospective | Serious | Serious | Moderate | Moderate | Serious | Moderate | Moderate | | Moderate |
| Mahmoud O et al. 2016 | Retrospective | Moderate | Moderate | Moderate | Low–Moderate | Moderate | Moderate | Moderate | | Moderate |
| Nakamura K et al. 2016 | Retrospective | Serious | Moderate | Serious | Moderate | Serious | Serious | Serious | | Serious |
| Song S et al. 2012 | Retrospective | Serious | Serious | Moderate | Serious | Serious | Moderate | Moderate | | Serious |
| Ryu SY et al. 2011 | Retrospective | Serious | Serious | Moderate | Serious | Serious | Moderate | Moderate | | Serious |
| Kim K et al. 2009 | Retrospective | Serious | Serious | Serious | Serious | Serious | Serious | Moderate | | Serious |
| Observation versus Chemotherapy | | |  |  |  |  |  |  | |  |
| Jiaxin F et al. 2025 | Retrospective | Serious | Serious | Moderate | Moderate | Serious | Serious | Moderate | | Moderate |
| Matsuo K, et al. 2024 | Retrospective | Moderate | Moderate | Moderate | Moderate | Moderate | Moderate | Moderate | | Moderate |
| Taguchi A et al. 2023 | Retrospective | Moderate | Moderate | Moderate | Moderate | Serious | Moderate | Moderate | | Moderate |
| Zhang J et al. 2022 | Retrospective | Serious | Serious | Serious | Serious | Serious | Moderate | Moderate | | Moderate |
| Nie J et al. 2021 | Retrospective | Serious | Serious | Serious | Moderate | Serious | Serious | Serious | | Serious |
| Li L et al. 2016 | Retrospective | Serious | Serious | Moderate | Moderate | Serious | Serious | Moderate | | Serious |
| Lee KB et al. 2008 | Retrospective | Serious | Serious | Moderate | Moderate | Serious | Serious | Moderate | | Serious |
